# Versatile John Allan Wyeth

**Published:** 1911-06

**Authors:** 


### EDITORIAL DEPARTMENT.

### VERSATILE JOHN ALLAN WYETH.

*"Ah, hard it is to climb the hill whereon Fame's proud temple shines afar."*

As an object lesson, a striking example of ambition, courage, pluck, relentless strenuosity, perseverance and determination to succeed, the early life and struggles of this great surgeon-author-teacher may be cited, for the benefit of his pupils and of all young men entering upon the study of medicine. No man ever climbed that hill over a road more difficult, dangerous and discouraging, nor reached its summit with greater *eclat* than this Alabama country boy. He fixed his standard high, and "went for it," despite every obstacle, and attained it. He is today the pride and boast of the true Southern physicians; that ideal being nothing less than the head of American medicine—President of the American Medical Association (1901-2), the representative of the entire medical profession of the United States of America.

These early experiences and struggles are chronicled in the Christmas number (1905) of the *Monthly Bulletin of the Arkansas Medical Society*—a small pamphlet. I wish I had room to reproduce the entire article; it should find permanent place in the libraries of the world, for it is a most unusual and remarkable document, autobiographic—which has the color of a phase in the evolution of our civilization—now happily past for ever,—the "reconstructive" days following the Civil War.

But I can not reproduce it in the little "Red Back"; it is too long. Believing, however, that some extracts from it—unconnected with his medical career—would interest my readers, I have selected and reproduce herewith one or two.

Dr. Wyeth hadn't a dollar when he was discharged from the Confederate ranks at the surrender. He went to work in Arkansas as a day laborer, saved his money, got a medical education, but learned to pilot a steamboat, and soon became owner, captain, pilot and roustabout of a small White River steamboat. He took a contract to get out the stone and build a jail in an Arkansas town, and was to get a bonus of a thousand dollars if he finished the job sixty days ahead of time. This is how he did it:

"Early in February, 1872, my work was so near completion that

I was sure to earn the bonus due if I should deliver the building by the first of May, provided I could get the one load of stone which was still at the quarry cut and ready for loading, awaiting water enough to float the barge over the shoals of the upper White. About this time I was encouraged to receive a telegram that a four-foot rise was coming from the mountains. I went post-haste to Jacksonport, and that night by 9 o'clock the 'Seminole,' from New Orleans, landed there on her way to Batesville for a barge of cotton. I called upon Captain Montgomery and asked him if he would tow my barge load of stone from Magness' quarry to Jacksonport. He flatly refused on account of the risk he would run in trying to handle a heavy barge in those dangerous waters. I told him of my contract and how important it was to me to get this material as far down at least as Jacksonport, whereupon he asked me if the barge was loaded and all ready. I replied 'No, but I will have it ready for you.' He said, 'It is now 9 o'clock. I expect to reach Batesville, take my cotton on board and by 12 o'clock tomorrow to be at your quarry on my way down the river. If you will have your barge loaded and ready by that time I will tow you to Jacksonport for $100.' I am of the opinion that he felt perfectly safe in making this offer, as he had not the remotest idea I could be ready for him.

"I shall never forget that ride! It was 10 o'clock when I went ashore from the 'Seminole.' The night was as black as Erebus. It was alternately sleeting and snowing and intensely cold for that region. On account of the darkness and the swift current which would impede her progress, I was satisfied I could beat the boat and save several valuable hours of time by going across the country on horseback. At the livery stable I asked for the best horse they had, and started for the ferry at the mouth of Black river. Arriving there, I was unable to get any response from the ferryman, who lived on the opposite shore and had the boat on that side. I hallooed at the top of my voice and fired my pistol twice in the direction of his cabin, but if he heard me he paid no attention. In all probability the cold and the darkness were enough to keep him in his warm bed. It was six miles up the river and out of my route to the next ferry, so I hastened there and fortunately found the ferryman's house on my side of the stream. By the offer of a considerable reward, he got out of bed and rowed me across. I still had to travel between twelve and fifteen miles of the worst roads imaginable through the White river swamp, with walls of cane on either side of the narrow way and with only two human habitations in this dismal stretch of country. At daylight I was on the bank of White river, opposite my destination. A native who had lived there and who had done considerable work for me came over with a canoe, and he and I went at once to the quarry to get the derrick in order. Meanwhile, I had sent couriers out for all the men in the neighborhood to come and help me, and to my great gratification, when at noon the 'Seminole' whistled around the bend above, the stone was all on board and I was ready.

"Billy Shipp, an old White River pilot, who not only knew the stream perfectly but the Mississippi from Memphis to New Orleans as well, and who had worked with me on the 'Converse,' was at the wheel on the 'Seminole' when I signaled. He told me afterwards of the conversation which occurred between Captain Montgomery and himself at that time. When he saw me on the deck of my barge waving my handkerchief to attract his attention, he remarked to the captain, who was standing by his side, 'There's the doctor, and he's ready for you.' Montgomery replied, 'What doctor?' Billy said, 'The young man who was talking to you last night at Jacksonport, and whose barge you agreed to tow out, provided he would have it ready for you when you came down today.' The captain said, 'Do you mean to tell me that that man rode up here last night and has that barge loaded!' Shipp responded, 'I do, and there he is,' whereupon Montgomery said, 'By God, if he lives, he will be President of the United States.' Captain Montgomery and I became warm friends after that day, but fortunately for me there was never the faintest hope of his prophecy becoming true. I had higher dreams than politics—dreams of a profession, the end and aim of which is and ever will be the betterment of the human race, and when, twenty-nine years after the date of which I am writing, I was elected President of the American Medical Association, the representative of the entire medical profession of the United States, I thought of this good friend of mine in my steamboat days in Arkansas, and only wished that he were living to know that I had gained (in my view of it) preferment beyond his prophecy, for I valued this honor higher than that of President of the United States.

"By the first of May my contract was finished, and I had earned the extra thousand dollars, and this, with what was still due me added to other savings, after paying all debts, left me with $4300 in cash, the net result of my three years in Arkansas. Few men have been happier than I when I realized that I now had sufficient means, by proper economy, to enable me to resume my medical education and to establish myself in the metropolis. Moreover, I had the good fortune while engaged in settling up the minor details of my affairs to dispose of the only barge left on my hands. A steamer, towing some craft, laden with locomotives for the railroad which was being built to the river at what is now Newport, struck a snag and sank, ten miles above Augusta. I went at once on horseback to the scene of the disaster and sold my barge at a good price to the captain in charge of the wreck. One feature of this short trip is impressed indelibly upon my memory.

"For two or three miles the route lay through slashes and swamp lands from which a big freshet had just receded and the road never much used was now not easy to trace, blocked as it was by logs and brush. On the way I had to cross a bayou about sixty yards wide, and for half of this distance my horse was floundering in the effort to swim. He became frightened, lost all sense of direction, and of course I could not guide him by the bridle, as any pull upon the rein of a swimming horse submerges him. I had no other recourse than to quit the saddle, hold on to his mane, swim with him and guide him by slapping him on one side of his head and then the other, in the direction of the roadway on the opposite side. Once over and knowing I had to return, I did not care to repeat this experience, and so I hallooed to a farmer who was working in a field nearby; told him that I would return in an hour or two, and asked him to have a skiff ready to take me over and swim the horse alongside, which he agreed to do. When I struck the road at the top of the bank there was some two miles further to go, and on account of the myriads of mosquitoes and gnats which attacked the horse and myself I was compelled to go at a rapid gallop in order to be rid of them.

"On the return, as I neared the bayou, I shouted at the top of my voice to the farmer to hurry up with the boat, for I knew these insects would run the horse crazy and might kill him if we tarried long. When I reached the edge of the bayou my man was not in sight, and in all probability had gone home to his dinner, as it was now midday. The moment I pulled the rein, clouds of ravenous insects attacked both horse and rider. As I was wearing gloves and had already fastened a large handkerchief around my neck and over my face, I was well protected; so hoping that I could fairly shield the horse until I could make the farmer hear me by loud hallooing, I dismounted, cut some brush and endeavored to fight them off. My task was as hopeless as Pharaoh's battle against the locusts in Egypt. They swarmed into the animal's ears and nostrils until he became frantic and unmanageable and tried to break away. No alternative was left but to mount and plunge into the water and take the risk of drowning or having him killed by these merciless insects. The home instinct of the animal served him better on the return trip, and he swam directly across. At a stiff pace, we were soon on the high grounds and had left our pests behind us. Those who have not lived in the White river lowlands in the summer season can not appreciate the terror and destructiveness of the insect life of that section. More numerous and more dangerous than the mosquitoes was the gnat or fly, called by some the 'Curtis' gnat, because it was said to have been introduced into that country with the army commanded by General Curtis when he invaded Arkansas during the Civil War. Others termed it the buffalo gnat on account of a peculiar hump upon its back. This insect came usually early in June and lasted for two or three weeks. It was so destructive to animal life that stock owners were compelled to keep a smudge going in their lots during the prevalence of the pest in order to prevent the destruction of their cattle. One farmer of my acquaintance lost seven horses and mules one night, and I have been assured by persons not given to exaggeration that they have seen wild deer quit their haunts in the swamps and seek refuge by leaping over fences and mingling with the cattle around the smoking fires. The fatal cases were due to suffocation, the gnats swarming into the nostrils and ears and arresting respiration."

Dr. Wyeth as a Fisherman.—I give another extract which, though somewhat irrelevant, is appropriate to the season. The dogwood is in bloom and the fish are biting fine. Somehow, as Tennyson says, "In the springtime the young man's fancy lightly turns to thoughts of love," while the old man's fancy lightly turns to the thoughts of going fishing. It was while the little boat was grounded on a sandbar and had to wait for a rise to float it, the doctor "went a fishin'." He says:

"On one occasion I landed three catfish at one and the same time with a single hook and line. One of these, about five inches long, I had placed on the hook as a bait. A second fish, sixteen inches in length, swallowed the bait and hook, and a third catfish, weighing thirty-eight pounds, swallowed the second fish head foremost, and finding he could not get off with his prey tried to eject it. In so doing the powerful lateral fins of the second fish were thrown out and impaled him. One of the fins had penetrated the abdominal walls of the large fish and was projecting fully a half inch beyond the skin. As the hook was fast in the stomach of the second fish this was the only way in which the larger animal could have been trapped, and when I ran the 'trot line,' from the strength he displayed in his efforts to break away and from the fact that the head and anterior portion of the second fish were not at all digested in the larger fish's stomach, I was convinced that he had just been caught. Joe Glover (who was in the boat with me at the time) and I could scarcely believe our eyes as I pulled these fish into the boat, but there they were and a dissection which I made revealed the three in one.

"It is not without some misgivings that even in the house of my friends I tell this story. It seems to be generally conceded that there is a natural elasticity in the web of truth in statements made by the followers of Izaak Walton. From the days of Jonah, 'fish stories' have been associated with the children of Ananias and Sapphira, and Burns must have had some piscatorial experience in mind when he wrote

'Some are lies frae end to end,
And some great lies were never penned;
Ev'n ministers, they ha'e been kenned,
In holy rapture,
A rousing whid at times to vend,
And nail 't wi' Scripture.'

"Fully twenty years after this occurrence I accidentally met my old friend Captain Glover, who was then a popular steamboat man on the Tennessee river, and we spent several enjoyable hours in the reminiscences of our Arkansas life, and in the course of our conversation he asked me if I had forgotten our catfish experience. To my reply that I had not, he asked me if I had ever told it. I said 'only in my own family or to my very intimate personal friends.' His rejoinder was, 'That is where you have shown your wisdom. In a moment of forgetfulness, I told that story to a gathering of men on my steamboat as we sat around the stove on a winter's night. I told it exactly as it occurred, and not a soul believed it.'"

